# Taxonomy and systematics are key to biological information: *Arabidopsis, Eutrema (Thellungiella), Noccaea* and *Schrenkiella* (Brassicaceae) as examples

**DOI:** 10.3389/fpls.2013.00267

**Published:** 2013-07-31

**Authors:** Marcus A. Koch, Dmitry A. German

**Affiliations:** ^1^Department of Biodiversity and Plant Systematics, Center for Organismal Studies Heidelberg, Heidelberg UniversityHeidelberg, Germany; ^2^South-Siberian Botanical Garden, Altai State UniversityBarnaul, Russia

**Keywords:** *Arabidopsis halleri*, *BrassiBase*, *Eutrema*, knowledge database, *Noccaea caerulescens*, *Schrenkiella*, taxonomy, *Thellungiella*

## Abstract

Taxonomy and systematics provide the names and evolutionary framework for any biological study. Without these names there is no access to a biological context of the evolutionary processes which gave rise to a given taxon: close relatives and sister species (hybridization), more distantly related taxa (ancestral states), for example. This is not only true for the single species a research project is focusing on, but also for its relatives, which might be selected for comparative approaches and future research. Nevertheless, taxonomical and systematic knowledge is rarely fully explored and considered across biological disciplines. One would expect the situation to be more developed with model organisms such as *Noccaea, Arabidopsis, Schrenkiella* and *Eutrema* (*Thellungiella*). However, we show the reverse. Using *Arabidopsis halleri* and *Noccaea caerulescens*, two model species among metal accumulating taxa, we summarize and reflect past taxonomy and systematics of *Arabidopsis* and *Noccaea* and provide a modern synthesis of taxonomic, systematic and evolutionary perspectives. The same is presented for several species of *Eutrema* s. l. and *Schrenkiella* recently appeared as models for studying stress tolerance in plants and widely known under the name *Thellungiella*.

*Noccaea caerulescens* (J. Presl and C. Presl) F. K. Mey. and *Arabidopsis halleri* (L.) O'Kane and Al-Shehbaz are the two top model-species among the Brassicaceae to study the ecology, physiology, molecular basis and evolution of metal stress and accumulation (e.g., Koch et al., 1998; Assunção et al., 2003; Clauss and Koch, 2006; Milner and Kochian, 2008; Krämer, 2010; Meyer and Verbruggen, 2012) (Figure 1). These species are indeed living in extreme environments, which is a phenomenon very often found in the Brassicaceae family. Another such group of species adapted to environments with high salt concentrations is known under the generic name *Thellungiella* O. E. Schulz. These “extremophytes” (Bressan et al., 2001; Inan et al., 2004; Amtmann, 2009) are widely used as a model for studying high salt resistance, drought and cold tolerance mechanisms in higher plant species.

**Figure 1:**
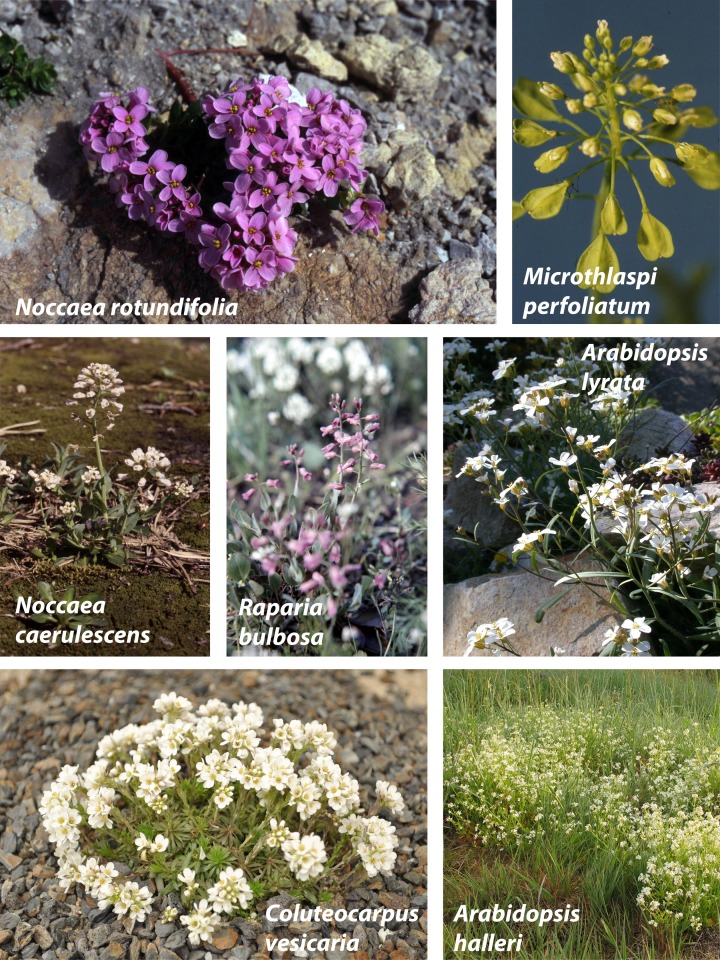
**Various representatives from the tribes Coluteocarpeae (*Noccaea, Raparia, Coluteocarpus, Microthlaspi*) and Camelineae (*Arabidopsis*)—photographs: M. A. Koch**.

All these taxa are, unfortunately, excellent examples to illustrate the lack of synergy across different biological disciplines (systematics/phylogeny on one hand and physiology, ecology, evolutionary biology, genetics and genomics, on the other). Information from these different disciplines is not cross-referenced; meaning systematic and taxonomic progress during the past decade(s) is being largely ignored. The enormous potential for comparative population and multiple-species based approaches is thus rarely fully explored. These species are discussed frequently within a misleading phylogenetic and evolutionary context and this applies to published data, which is still being cited without any (corrective) commentary.

In the following paragraphs we show that the taxonomic recognition of the genus *Noccaea* Moench and thereby *Noccaea caerulescens* is largely incorrect and frequently ignored by physiologists and molecular biologists. Instead “*Thlaspi*” is widely used. Moreover, we are referring here to four decades of research history (not limited to a few years). In contrast, biologists have been much more amenable to the use of any *Arabidopsis* (DC.) Heynh. taxon (terminology) during the same time period, presumably considering the genus as containing the “closest” relatives of the model plant *Arabidopsis thaliana* (L.) Heynh. (Clauss and Koch, 2006). Consequently, it was probably easier to educate scientists to work with particular “*Arabidopsis*” species, even if they are not closely related to the taxonomic “beacon” (*Arabidopsis thaliana*). *Thellungiella* represents another and probably the most acute of the discussed cases of a particular lack of interdisciplinary connections. Similar to *Noccaea*, a widely applied concept of *Thellungiella* in non-taxonomic literature generally ignores the state of the art of taxonomy and phylogeny of affected taxa. And in many cases systematics and taxonomy is wrongly used at species, generic, and tribal level with relevant consequences in the interpretation of the results. However, this incongruence has a much shorter history (though higher degree) than in *Noccaea*, and there is a chance to overcome this situation much faster. Our intention here is to present and discuss these issues in more detail and to illustrate the value of taxonomy and systematics as a bioinformatics tool (Koch et al., 2012).

## Taxonomic history, biogeography and systematics of *Arabidopsis, Noccaea* and *Thellungiella*/*Eutrema*

### The life and (hard) times of the genus *Noccaea*

Various species belonging to the genus *Noccaea* have traditionally been treated under a broadly defined genus *Thlaspi* L. This genus was originally described by Linnaeus (1753), and it should not be surprising that a 250 year-old generic concept, though undergoing various updates, does not reflect any real or meaningful phylogenetics. Much later, Meyer (1973, 1979) revised the generic concept based mostly on seed coat anatomy and embryology, and placed many of the former *Thlaspi* species into a well-defined genus *Noccaea*. As indicated by the authority Moench, Meyer was not the first who recognized this genus, but Moench did so in (1802) by recognition of the species, previously known as *Iberis rotundifolia* L. or *Lepidium rotundifolium* (L.) All. (and later widely accepted as *Thlaspi rotundifolium* (L.) Gaudin), as a single member of *Noccaea* thus making *Noccaea rotundifolia* the type of the genus, but also ignoring many other species to be integrated into *Noccaea*. Furthermore, Meyer did not only recognize the genus *Noccaea*, but he introduced also eleven additional new genera to newly combine various *Thlaspi* s. l. species into such genera as *Raparia* F. K. Mey., *Microthlaspi* F. K. Mey. and others.

**Figure 2:**
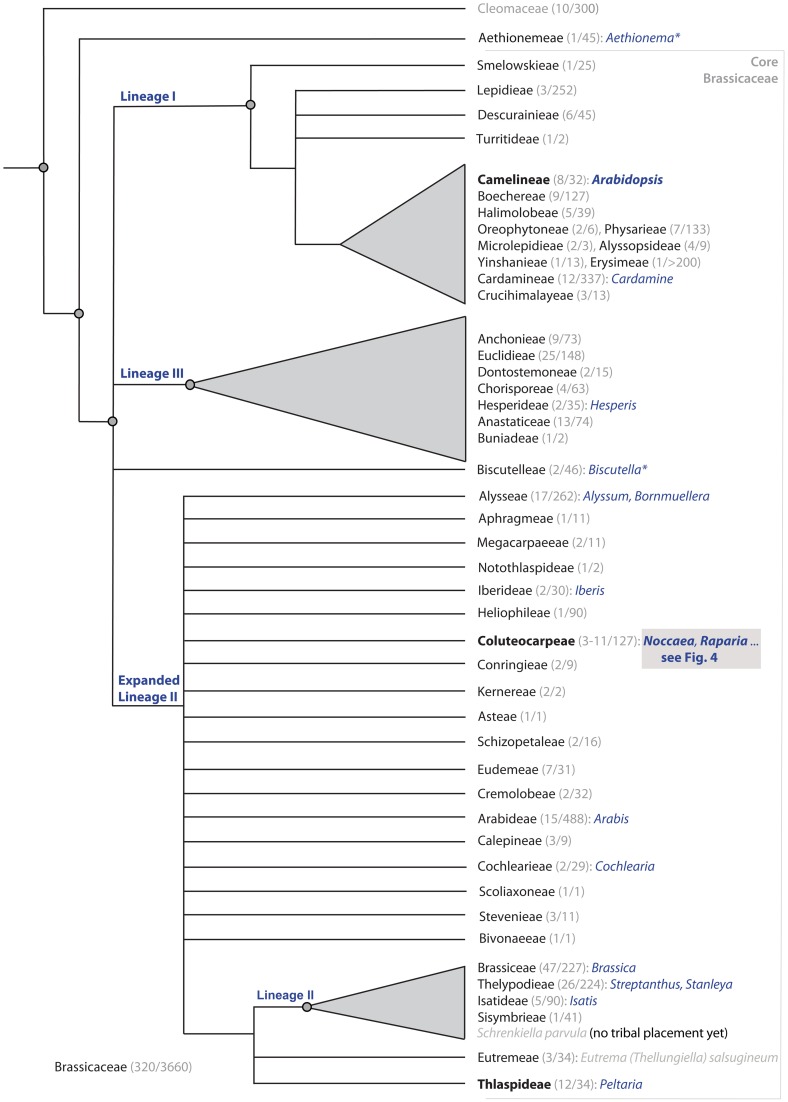
**Cartoon on phylogenetic relationships of the Brassicaceae among tribes, with given numbers of genera and species respectively (for detail, Koch et al., 2012).** Tribes with species showing the trait of metal accumulation are indicated with some example genera named in blue (taken from Krämer, 2010; with several corrections of tribal affiliation). Tribes comprising the genus *Arabidopsis* (Camelineae), *Noccaea* and its relatives (Coluteocarpeae) and the few remaining *Thlaspi* species (Thlaspideae) are indicated in bold/black. Genera marked with an asterisk do also comprise species occurring frequently on metal rich soil types.

It is remarkable that this taxonomic solution would have also combined most of the metal hyperaccumulator species of the former “*Thlaspi*” into one monophyletic group. However, Meyer's concept still assumes close relationships between different former “*Thlaspi*” segregates, which turned out later to be wrong. Molecular systematic studies (Mummenhoff and Koch, 1994; Mummenhoff et al., 1997a,b) in general confirmed Meyer's generic concept, but it was also demonstrated that some of the segregates are not closely related to each other. In particular, *Thlaspi* sensu Meyer (generic type *T. arvense* L.) is not at all related to *Noccaea* (Figure 2), and the two genera are placed in two different and not related tribes (Al-Shehbaz et al., 2006; Couvreur et al., 2010; Franzke et al., 2010).

**Figure 3:**
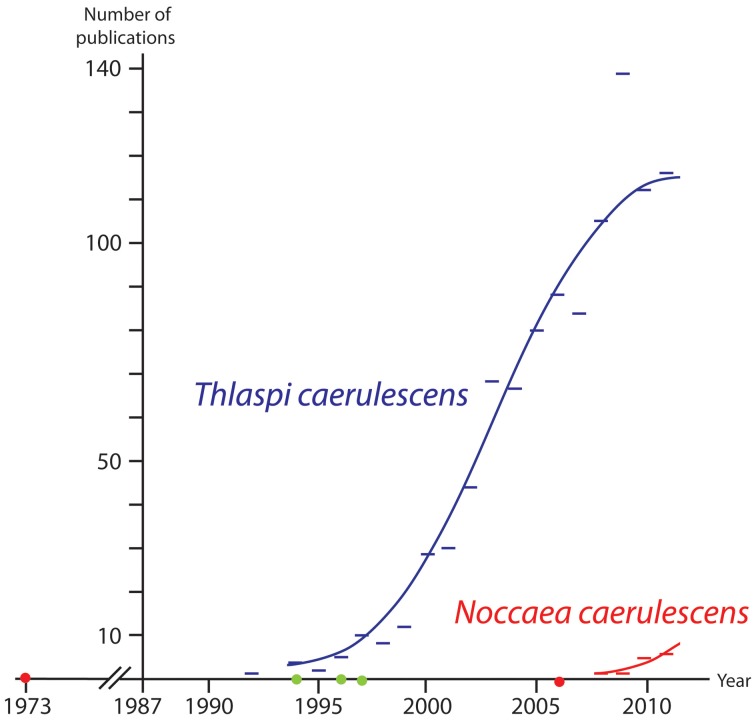
**Results of a Web of Science search (March 2nd 2012) from 1987 (indexing starting point) to 2011.** Publications indicated by a blue bar includes the term “*Thlaspi caerulescens*” (out-dated taxonomical treatment) in their title, abstract or keywords and not the term “*Noccaea*”. Publications indicated by a red bar include the term “*Noccaea caerulescens*” (current reasonable treatment) in title, abstract or keywords. Red dots: Meyer re-established the genus *Noccaea* in 1973 and provided further details in 2006. The green dots indicate publication years of early molecular studies supporting Meyer's concept: Mummenhoff and Koch (1994); Zunk et al. (1996); Mummenhoff et al. (1997a,b).

Based on morphological and particularly anatomical characters, Meyer (1973, 1979) (correctly) recognized *Noccaea* as the well-defined group. Twenty years later, molecular evidence which confirmed this finding from a phylogenetic point of view was published (Mummenhoff and Koch, 1994; Zunk et al., 1996). But what happened subsequently, in the 10 years that followed? The new taxonomy was again neglected or overlooked by physiologists, ecologists and evolutionary biologists. Since Meyer started working on the genus *Thlaspi* sens. trad. in the early 1970s, he continuously published a series of monographs of all his new genera. A comprehensive volume focusing on *Noccaea* was presented by Meyer in (2006). Additional work has been presented confirming *Noccaea* as a distinct genus (Koch and Mummenhoff, 2001; Koch and Al-Shehbaz, 2004). However, most of the scientific contributions focusing on the model organism *Noccaea caerulescens* ignored these achievements and did not provide the relevant links (mentioned initially by Koch et al., 1993). There are various reasons that can explain this situation. One reason might be that almost all Meyer's works were published in German and in purely taxonomic journals. We tried to count the number of publications in the last 25 years indexed in the ISI Web of Science focusing on *Noccaea/Thlaspi caerulescens* any using one or the other taxonomic option (Figure 3; redrawn from Koch et al., 2012). The implication from this figure appears to be that more than three decades were required to disseminate according knowledge to a broader community which has only gradually moved toward using the combined wealth of information from taxonomy and wider disciplines (comparative biogeography, evolutionary history, trait and characters such as metal hyperaccumulation).

Since *Noccaea* comprises the vast majority of species diversity within *Thlaspi* sensu trad., which is well-known as a taxonomically complex entity, most of those problems are now connected with *Noccaea*. Neither generic limits, grouping within the genus, nor the limits and the relationships of a number of species have been finally resolved. The species richness estimates of *Noccaea* range considerably—from ca. 85 (Al-Shehbaz et al., 2006) to 120 (Al-Shehbaz, 2012). Phylogenetically, the best studied group is the North American *Noccaea* species complex (Koch and Al-Shehbaz, 2004), and only a limited group of a few European *Noccaea* species fall into well-supported clades (Koch et al., 1993; Mummenhoff and Koch, 1994).

**Figure 4:**
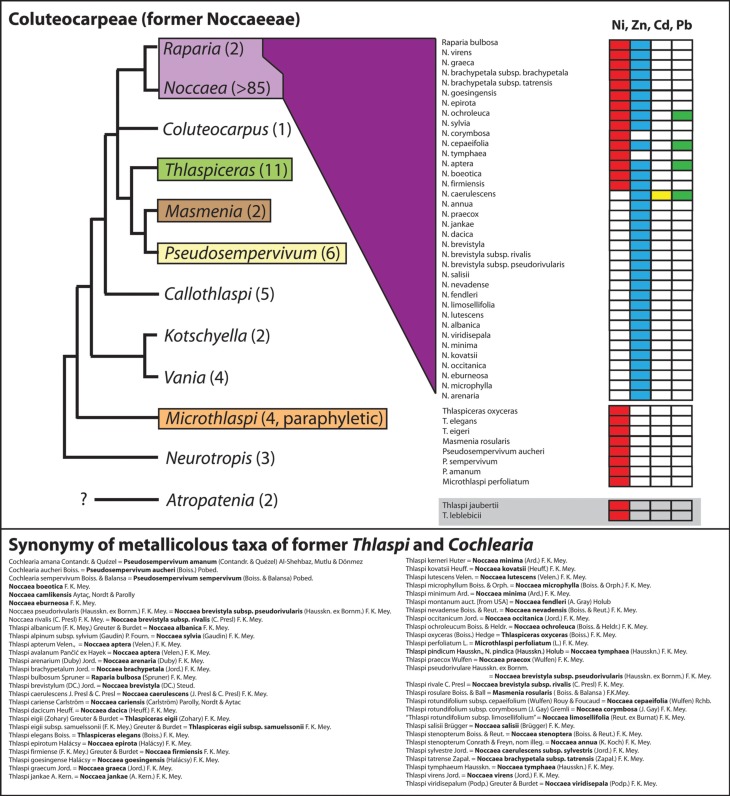
**Cartoon on phylogenetic relationships within the tribe Coluteocarpeae (syn. Noccaeeae) and the distribution of metallicolous taxa.** This tribe comprises the majority of the many taxa previously combined under a broadly defined genus *Thlaspi* but also few former *Cochlearia* species. The phylogenetic hypothesis is based on DNA sequence data of the Internal Transcribed Spacers 1 and 2 of nuclear ribosomal DNA (Koch et al., 2012). Taxa have been considered as accumulators of nickel, cadmium, lead or zinc, respectively, if reports are available with the metal concentration in leafs exceeding thresholds as reviewed in Krämer (2010) (nickel and lead: 1000 μg/g dry-mass; zinc: 10,000 μg/g dry-mass; cadmium: 100 μg/g dry-mass). The compilation of taxa follows Brooks et al. (1977), Reeves (1988); Reeves and Brooks (1983); Reeves et al. (2001); Adigüzel and Reeves (2012), and see Reeves and Baker (2000) for a detailed review. Note: Correct name for this tribe is Coluteocarpeae (Dorofeyev, 2004) and not Noccaeeae (see Al-Shehbaz, 2012). For *Atropatenia* no ITS sequence data are actually available. Phylogenetic position and systematic affiliation of *Thlaspi jaubertii* Hedge and *Thlaspi leblebicii* Gemici and Görk are currently unclear. Total species number for the different genera is given in brackets.

Among the various *Noccaea* species well-known (and intensively studied) for their occurrence on metal rich habitats are European *N. goesingensis* (Halácsy) F. K. Mey., *N. montana* (L.) F. K. Mey., *N. praecox* (Wulfen) F. K. Mey., *N. tymphaea* (Hausskn.) F. K. Mey. (sometimes reported as *Thlaspi pindicum* Hausskn.) and *N. sylvia* (also known as *Thlaspi alpinum* subsp. *sylvium*) (e.g., Vogel-Mikus et al., 2005; Taylor and Macnair, 2006) or North American *N. fendleri* (as *Thlaspi montanum* in Boyd and Martens, 1998) (for a most comprehensive review, see Reeves and Baker, 2000). Many other metallicolous species found with the generic designation *Thlaspi*/*Noccaea* are listed in Figure 4 and the best current reconstructed taxonomy is provided. In total 15 nickel accumulator and 32 zinc accumulator species have been described from the genus *Noccaea* and closely related genera [*Masmenia* F. K. Mey., *Pseudosempervivum* (Boiss.) Grossh.]. A few of them also accumulate cadmium and lead.

*Noccaea caerulescens* is among the most variable and taxonomically difficult species of the genus (see, for instance, Jalas et al., 1996, as *Thlaspi caerulescens* J. Presl and C. Presl). Its synonymy includes a number of species epithets (Meyer, 2006) and several (collectively up to seven only in the recent publications) subspecies are recognized by different authors (e.g., Clapham and Akeroyd, 1993; Jalas et al., 1996; Holub, 1998; Dvořáková, 2003; Meyer, 2006; Marhold, 2011). An additional complication is caused by differences in the attribution of the widely used illegitimate name *Thlaspi alpestre* L. (1763, non Jacq., 1762) which has long been and is still treated conspecific with either *N. caerulescens* (Cafferty and Jarvis, 2002) or closely related *N. brachypetala* (Jord.) F. K. Mey. (Meyer, 2006) which itself is treated by some authors as a subspecies of *N. caerulescens* [as *T. caerulescens* subsp. *brachypetala* (Jord.) O. Bolòs, Vigo, Masalles and Ninot] (e.g., Jalas et al., 1996), though the latter viewpoint is not generally followed. According to Meyer (2006), recent typification of the name *T. alpestre* L. (Marhold and Martonfi in Cafferty and Jarvis, 2002) finally brought it to synonymy of *N. brachypetala* which should have stopped taxonomic confusions connected with this Linnean binominal. However, it does not remove another problem further contributing to the complexity of nomenclature of this group, namely, persisting differences in the interfering concepts of *N. caerulescens* and *N. brachypetala* among taxonomists. Quite widely accepted, is a viewpoint separating the species morphologically primarily by petal length (1–1.5 mm long, equaling or up to 1,3 times exceeding sepals in *N. brachypetala* versus. 2–4 mm long and 1.5–3 times exceeding sepals in *N. caerulescens*) (e. g., Clapham and Akeroyd, 1993; Pujados Salvá, 1993, both as *Thlaspi*). This sufficiently differs from Meyer's approach “allowing” *N. brachypetala* to have petals 0.8–3 mm long. As a result, the limit between the two entities is not obvious, and a number of names of taxa described predominantly from central and eastern France are treated as synonyms of either *N. caerulescens*, or *N. brachypetala* (conf. Meyer, 2006 vs. Marhold, 2011), or as subspecies of any of them. Meyer (1973, 2006) accepted species status of *N. brachypetala* (with three subspecies) and, unlike other authors (e. g., Dvořáková, 2003) accepted only two subspecies of *N. caerulescens*: subsp. *caerulescens* and subsp. *sylvestris* (Jord.) F. K. Mey.; synonymy of the latter includes, in particular, *T. calaminare* (Lej.) Lej. and Courtois.

The above mentioned discrepancies along with the fact that Meyer's (1973, 1979, 2006) circumscription of *Noccaea*, including the recognition of two subspecies of *N. caerulescens* and his generic concept has not been followed in recent floristic treatments in Europe, it is difficult to unravel putative distribution ranges and occurrence of the various taxa. For example, Meyer does not confirm previous reports of *N. caerulescens* for Spain (e.g., Clapham and Akeroyd, 1993; Pujados Salvá, 1993 as *Thlaspi*). Therefore, we provide a table showing the most important synonyms with their respective geographical source (Table 1) and based predominantly on Meyer's approach (Meyer, 2006). However, it should be noted here that we are missing substantial molecular, phylogeographic data linking taxonomy with evolutionary history in space and time. The few studies done so far can be summarized shortly. A study of limited resolution and using isoelectric focusing of the various subunits of the RuBisCO protein highlighted some closer relationships, especially between *Noccaea caerulescens* and *N. brachypetala* (Koch et al., 1993; nota: here also the old *Thlaspi* synonymy was used, but discussed within Meyer's framework). This closer affinity was later confirmed by DNA based markers (Mummenhoff and Koch, 1994). A more comprehensive study based on isozyme analysis and focusing on various populations of *Noccaea caerulescens* and *N. brachypetala* from contaminated and non-contaminated sites was presented later (Koch et al., 1998) demonstrating that the *N. caerulescens* ecotype accumulating metals might have evolved several times independently. There is also increasing ecological knowledge of the various populations of *N. caerulescens*, and it has been shown, for example, that there are significant differences in life-history traits (Macnair, 2007; Dechamps et al., 2011), in outcrossing rates (Koch et al., 1998; Duboix et al., 2003), or in the strength of natural selection limiting gene flow between metalliferous and non-metalliferous locations (Besnard et al., 2009).

**Table 1:** ***Noccaea caerulescens* and *N. brachypetala* synonymy and distribution including geographic source of respective type material**.

**Name**	**Type locality**	**Synonym of (following Meyer, 2006)**	**Distribution (following Meyer, 2006)**
*Thlaspi caerulescens* J. Presl and C. Presl [*T. alpestre* subsp. *sylvestre* f. *caerulescens* (J. Presl and C. Presl) Thell.; *T. alpestre* var. *caerulescens* (J. Presl and C. Presl) Domin]	Czech Republic (Bohemia)	*Noccaea caerulescens* (J. Presl and C. Presl) F. K. Mey. subsp. *caerulescens*	Czech Republic, Poland, Germany, Austria
*T. alpestre* var. *pseudocalaminare* Domin	Czech Republic		Adventive: Norway, Sweden, Finland, NW Russia
*T. sylvestre* Jord. [*T. alpestre* subsp. *sylvestre* (Jord.) Nyman; *T. alpestre* var. *sylvestre* (Jord.) Bab.]	France (Lyon, Soucieux)	*Noccaea caerulescens* subsp. *sylvestris* (Jord.) F. K. Mey	Great Britain, Netherlands, Belgium, France, Germany, Switzerland, Italy
*T. alpestre* var. *calaminare* Lej. [*T. calaminare* (Lej.) Lej. and Courtois; *T. alpestre* subsp. *calaminare* (Lej.) O. Schwarz; *T. caerulescens* subsp. *calaminare* (Lej.) Dvořáková; *N. caerulescens* subsp. *calaminaris* (Lej.) Holub]	Belgium (Wallonia)		
*T. alpestre* var. *glareosum* Dumort., **nomen nudum!**	Belgium		
*T. gaudinianum* Jord. [*T. alpestre* subsp. *gaudinianum* (Jord.) Gremli; *T. sylvestre* f. *gaudinianum* (Jord.) Rouy and Foucaud; *T. caerulescens* subsp. *gaudinianum* (Jord.) Dvořáková]	W Alps (Jura mts.)		
*T. vogesiacum* Jord. [*T. sylvestre* var. *vogesiacum* (Jord.) Rouy and Foucaud]	France (Vogeses, Bussang)		
*T. ambiguum* Jord. [*T. sylvestre* var. *ambiguum* (Jord.) Rouy and Foucaud]	France (Vogeses)		
*T. alpestre* var. *porphyreum* Wirtg.	Germany		
*T. alpestre* var. *grandiflorum* Godr.	France (Lorraine)		
*T. sylvestre* var. *oligospermum* Merino [*T. oligospermum* (Merino) Greuter and Burdet; *N. oligosperma* (Merino) Holub]	NW Spain (Mellid)		
*T. brachypetalum* Jord. [*T. alpestre* var. *brachypetalum* (Jord.) Gremli; *T. alpestre* subsp. *brachypetalum* (Jord.) Thell.; *T. caerulescens* subsp. *brachypetalum* (Jord.) O. Bolòs, Vigo, Masalles and Ninot; *N. caerulescens* subsp. *brachypetala* (Jord.) Tzvel.]	France (W Alps) (Environs de Grenoble et de Gap, bois de la Grangette)	*Noccaea brachypetala* subsp. *brachypetala* (Jord.) F.K. Mey.	France, Spain, Switzerland, Italy, Austria Adventive: Finland, Sweden
*T. alpestre* L. (non Jacq.)	Austria		
*T. virgatum* Gren. and Godr.	France (W Alps and E Pyrenees)		
*T. lereschii* Reut. [*T. alpestre* subsp. *lereschii* (Reut.) Gremli; *T. sylvestre* var. *lereschii* (Reut.) Rouy and Foucaud]	Vicinity to Lac Léman, Jura mts.		
*T. vulcanorum* Lamotte [*T. brachypetalum* var. *vulcanorum* (Lamotte) Rouy and Foucaud]	Central France (Cantal)		
*T. arnaudiae* Jord.	Central France		
*T. suecicum* Jord. [*T. alpestre* subsp. *brachypetalum* var. *suecicum* (Jord.) Hyl.]	Sweden		
*T. salticorum* Jord. [*T. brachypetalum* var. *vulcanorum* f. *salticorum* (Jord.) Rouy and Foucaud]	France		
*T. verlotii* Jord. [*T. brachypetalum* var. *vulcanorum* f. *verlotii* (Jord.) Rouy and Foucaud]	France (W Alps, Isère)		
*T. nemoricolum* Jord. [*T. brachypetalum* var. *nemoricolum* (Jord.) Rouy and Foucaud]	France (Cantal)		
*T. brachypetalum* var. *costei* Rouy and Foucaud	France (Aveyron, Salles-Curan)		
*T. tatrense* Zapał. [*T. sylvestre* subsp. *tatrense* (Zapał.) Dvořáková; *T. alpestre* subsp. *tatrense* (Zapał.) Soó; *T. caerulescens* subsp. *tatrense* (Zapał.) Dvořáková]	Slovakia (Tatra mts., Krywañ)	*Noccaea brachypetala* subsp. *tatrensis* (Zapał.) F.K. Mey.	Czech Republic, Slovakia, Austria
*T. alpestre* var. *stenopterum* Borbás	South East Austria (Burgenland)		
*T. alpestre* var. *demissorum* Borbás	South East Austria (Burgenland)		
*T. huteri* Pernh. [*T. sylvestre* var. *huteri* (Pernh.) Dalla]	NE Italy (Gsies)	*Noccaea brachypetala* subsp. *huteri* (Pernh.) F. K. Mey.	Austria, Italy

At present we have no direct evidence and calculations for the timing of *Noccaea caeruelscens*' split from a common ancestor. But considering the low genetic variation found within and between populations compared to other Brassicaceae genera and comparing it also with the evolutionary scenario of American *Noccaea* species (Koch and Al-Shehbaz, 2004) it can be concluded that *N. caerulescens* is of a Pleistocene origin and diverged from their European relatives less than one million years ago.

### We list the following conclusions and provide suggestions for future research

1. The name *Noccaea caerulescens* should be used instead of *Thlaspi caerulescens* in any contribution providing access to the correct systematic, evolutionary framework. This should prevent the accumulation of data interpreted in the wrong phylogenetic context.
2. It is nearly impossible to find morphological characters that are clearly indicative of metallicolous ecotype(s) of *N. caerulescens*. Consequently, recognizing such plants as taxonomic entities of subspecies or species rank (e. g., subsp. *calaminare*) is not justified.
3. Following Meyer's concept, all metallicolous populations together with non-metallicolous populations should be taxonomically treated as *Noccaea caerulescens* subsp. *sylvestris* with a western Central European distribution which include populations from the United Kingdom. The eastern Central European vicariant is best treated as *N. caerulescens* subsp. *caerulescens*, which also colonized Scandinavia very successfully. However, future phylogeographic studies will have to demonstrate if this concept can be followed (completed with genetic data). Regardless, we can assume a largely overlapping distribution area with extensive admixture since the last glacial maximum.
4. It is not clear yet, how the different taxa of *N. brachypetala* (with the closest affinity) should be recognized. Among contrasting viewpoints, Meyer treats *N. brachypetala* as separate species with three subspecies mostly characterized by their occurrence at higher elevation from mountainous to alpine habitats (subsp. *brachypetala*, subsp. *tatrensis* (Zapał.) F. K. Mey., subsp. *huteri* (Pernh.) F. K. Mey.). When considering populations from France (e.g., Massif Central) or Spain, however, this concept might need to be revised.
5. It has also to be noted that few other taxa from Meyer's series *Alpestres* show very close affinities (geographically and taxonomically) with *N. caerulescens* and *N. brachypetala*: namely *Noccaea virens* (Jord.) F. K. Mey. (often treated also as a subspecies of *N. caerulescens*) and *N. salisii* (Brügger) F.K. Mey. In addition maybe also *N. occitanica* (Jord.) F.K. Mey. and its two subspecies from Meyer's series *Occitanicae* are within the *N. caerulescens*/*N. brachypetala* species aggregate.

### Life in the fast lane: the genus *Arabidopsis*

“*Arabidopsis* and its poorly known relatives”, this title was used recently for a review introducing the closest relatives of *Arabidopsis thaliana*, and all members of the currently defined genus *Arabidopsis* (Clauss and Koch, 2006). A major part of the taxonomic confusion arose after the year 1872 when many species were transferred into a genus *Arabidopsis* based on a few simple morphological characters (cf. latiseptate siliques and branched trichomes). As a result, some 60 species were recognized in *Arabidopsis* in a traditional sense (see Al-Shehbaz et al., 1999; German and Ebel, 2005). Major aspects of this *Arabidopsis*' taxonomical history were compiled in detail Al-Shehbaz et al. (1999); Al-Shehbaz and O'Kane (2002) and nine *Arabidopsis* species with several subspecies were recognized by this time. Note that Hedge (1968) indirectly suggested a closer relationship between *Arabidopsis* and *Arabis* L. He indicated that the two genera differ only in the position of the cotyledons relative to the radicle in the seeds and that the Himalayan species *Arabidopsis wallichii* (Hook. f. and Thomson) N. Busch is essentially intermediate between the two genera. However, as molecular data were lacking, Hedge and others were not aware that, in fact, the genus *Arabis* at that time comprised several unrelated evolutionary lineages (see Koch et al., 1999, 2000, 2001; Karl and Koch, 2013). In addition, the historical concept of *Arabis* as a genus does not exist anymore (Koch et al., 1999, 2001; Karl and Koch, 2013). Because taxa from both genera, *Arabidopsis* and *Arabis*, share a taxonomic history, they frequently exhibit related taxonomic nomenclatural problems with numerous misleading phylogenetic implications. See also the taxon *Arabidopsis wallichii*, which Hedge suggested to be an intermediary between *Arabidopsis* and *Arabis*, and which has now been christened *Crucihimalaya wallichii* (Hook. f. and Thomson) Al-Shehbaz, O'Kane and R. A. Price and is not closer related to any of these genera phylogenetically.

The new and currently accepted concept of the genus *Arabidopsis* was presented 10–15 years ago (O'Kane and Al-Shehbaz, 1997, 2003), in parallel by a contribution from Koch et al. (1999) who unraveled some taxonomical problems including both *Arabis* and *Arabidopsis*. Some species and subspecies were added later, however, either because of a transfer of taxa previously never associated with *Arabidopsis* (Warwick et al., 2006), or a description of a new species (Kadota, 2007), Mostly, the delay was due to raising the rank of some other taxa treated by O'Kane and Al-Shehbaz (l. c.) as subspecies or synonyms (Dorofeyev, 2002; Marhold et al., 2003; Shimizu et al., 2005; Kolník and Marhold, 2006; Iljinska et al., 2007; Kadota, 2007; Elven and Murray, 2008). In summary, in many cases it reflected the delimitations of previous authors. As a result, depending on the approach, *Arabidopsis* can be estimated as a genus comprising at least nine species and six subspecies (O'Kane and Al-Shehbaz, 1997), up to 13 species and nine subspecies (e.g., summarized in Koch et al., 2008).

It is not only *A. halleri* that grows on metal rich soils, but initial work has been also done on North American *A. lyrata* (L.) O'Kane and Al-Shehbaz from serpentine soils (Turner et al., 2008, 2010). And also in Eastern Austria some populations of *A. lyrata* are geographically close to serpentine outcrops (Schmickl and Koch, 2011).

Delimitation of *Arabidopsis halleri* is not congruent among taxonomists. Up to five subspecies can be recognized (O'Kane and Al-Shehbaz, 1997, 2003; Kolník and Marhold, 2006; Koch et al., 2008) though two of them, *A. halleri* subsp. *gemmifera* (Matsum.) O'Kane and Al-Shehbaz and *A. halleri* subsp. *ovirensis* (Wulfen) O'Kane and Al-Shehbaz are accepted by some authors as separate species, *A. gemmifera* (Matsum.) Kadota and *A. ovirensis* (Wulfen) A. P. Iljinsk., respectively (Kadota, 2007; Iljinska et al., 2007). Taxonomic treatment of Kolník and Marhold (2006) recognizes three predominantly Central European subspecies: most common subsp. *halleri* (latitudinally from Poland to Italy and Serbia and longitudinally from Belgium and France to W Ukraine and Moldova; substrate-indifferent, ranging from foothills to alpine belt), subsp. *tatrica* (Pawł.) Kolník (W Carpathian endemic, almost completely confined to Slovakia; substrate-indifferent, ranging from foothills to alpine belt), and subsp. *dacica* (Heuff.) Kolník (E and S Carpathians [Romania], probably somewhat further southwards into the Balkans; restricted to acid substrata and predominantly alpine, rarely montane habitats). And, indeed, E Asian *A. halleri* subsp. *gemmifera* is not only genetically separated from the other subspecies (Koch et al., 2008), but also geographically fully isolated. *Arabidopsis halleri* subsp. *ovirensis* has been originally described as endemic to the East Austrian high mountain range at Mount Obir, in Carinthia. Reports from other localities (e.g., from Romania and Ukraine) need confirmation. Genetic results showing some unique genetic markers in these respective populations are in agreement with this endemic distribution (Koch and Matschinger, 2007; Koch et al., 2008).

In the case of *Arabidopsis halleri* we have some more detailed evidence for its evolutionary history. It has been shown that all five above mentioned subspecies are closely related to each other, and that one major center of genetic diversity is located in the Eastern Austrian Alps (Koch and Matschinger, 2007). Very similar to *N. caerulescens* (Koch et al., 1998), it has also been concluded for *A. halleri* that metallicolous populations have been founded separately from distinct non-metallicolous populations without suffering founding events (Pauwels et al., 2005). This study was exclusively focused on *A. halleri*, and did not mention any further subspecies. However, it is likely that the authors included one sample of *A. halleri* subsp. *tatrica* with a distinct chloroplast haplotype not found in the remaining Central European populations. A comprehensive phylogeographic scenario was presented recently (Pauwels et al., 2012), and although the accessions studied were again not characterized taxonomically, many helpful comments linking taxonomy with genetic evidences were provided. High levels of genetic diversity found in the eastern region of the European Alps and initially demonstrated by Koch and Matschinger (2007) were confirmed and explained convincingly by admixture and secondary contact of different European gene pools.

With similar parallels to *Noccaea caerulescens*, the evolutionary scenario of *A. halleri* is best placed among Pleistocene glaciation and deglaciation cycles (Koch and Matschinger, 2007). In a more detailed study, Roux et al. (2011) suggested the onset of radiation within *A. halleri* to be 335,000 [272,800–438,200] years ago, but because this study lacks other subspecies, a deeper evolutionary split is possible.

In contrast to *Noccaea caerulescens,* and bearing in mind the detailed investigations in *Arabidospis* species (e.g., *A. thaliana* and *A. lyrata*), the number of genetic-evolutionary studies focusing on *Arabidopsis halleri* is high (e.g., Van Rossum et al., 2004; Meyer et al., 2009; Heidel et al., 2010). Numerous ecological studies are of course also available focusing on herbivory (Kawagoe and Kudoh, 2010), flowering time (Shimizu et al., 2011) or reproduction (Llaurens et al., 2008), for example.

### As with *Noccaea Caerulescens*, we provide some conclusions and recommendations

1. Within *A. halleri* there are three to five different subspecies (*gemmifera, tatrica, halleri, ovirensis*, and *dacica*), of which *A. halleri* subsp. *ovirensis* is a genetically distinct endemic taxon. Thus, *A. halleri* subsp. *ovirensis* and geographically isolated *A. halleri* subsp. *gemmifera* could be treated as species, but it would be difficult to present convincing morphological evidence to recognize them accordingly, which should be a pre-requisite prior to any further taxonomical changes.
2. The metal accumulating and metallicolous populations characterized so far are mostly from *A. halleri* subsp. *halleri*. However, zinc and cadmium accumulating populations have been characterized also within *A. halleri* subsp. *gemmifera* (Kubota and Takenaka, 2003; Kashem et al., 2007) and occurrence of subsp. *tatrica* on metal-contaminated soils has been reported (Kolník and Marhold, 2006).

### *Thellungiella*: an emerging model system which remains taxonomically challenging

*Thellungiella* O.E. Schulz is gradually becoming established as a new (Arabidopsis-like) extremophyte model ideal for studying salt, drought and cold tolerance (beginning some 12 years ago, Bressan et al., 2001). During this period, it has become an established model system (see thellungiella.org) and complete genomes of two *Thellungiella* species, *T. salsuginea* (Pall.) O.E. Schulz and *T. parvula* (Schrenk) Al-Shehbaz and O'Kane, are now available (Dassanayake et al., 2011; Wu et al., 2012) providing a resource for deep insights into the evolutionary mechanisms underlying stress tolerance and various other physiological processes.

Since the advent of these genomic resources, a considerable amount of “simple” but basic information regarding *Thellungiella* taxonomical diversity, phylogeny and geographical range has accumulated which has been (and still is) heavily neglected. As a result, the current concept of *Thellungiella* in these studies implies polyphyly and a mixture of up to three species. This has serious negative consequences: a source of rapidly proliferating misinterpretations and even artifacts (when the functions of one biological species are attributed to another).

In 2001, when the review of Bressan et al. (2001) was published, *Thellungiella* was treated as a genus of three species, *T. salsuginea, T. parvula* and *T. halophila* (C. A. Mey.) O.E. Schulz (Zhou et al., 2001) and their phylogenetic position and relationships were rather unclear. As evidenced from studies of stress tolerance, the name *T. halophila* was applied wrongly (right from the start). This is perfectly illustrated by the following sentence: “*Thellungiella halophila*, previously classified as *Arabidopsis halophila*, recently has been reclassified as *Thellungiella salsuginea* (Al-Shehbaz et al., 1999), which now can be considered synonymous with *Thellungiella halophila* (salt cress)” (Inan et al., 2004). In fact, the latter species is not mentioned in Al-Shehbaz et al. (1999), and this obvious confusion most likely has its roots in an over-interpretation of the earlier data of Al-Shehbaz and O'Kane (1995) who suspected possible (but did not absolutely claim) conspecificity of *T. halophila* with *T. salsuginea*. These authors did not make a formal synonymization of *T. halophila* and recognized *Thellungiella* as a “genus of two (or perhaps three [i. e., *T. halophila, T. parvula* and *T. salsuginea*]) species” thus keeping the question open till the study of the type material of *T. halophila*. Later, distinctness of the two discussed species was confirmed (Zhou et al., 2001), but the practice of using the name *T. halophila* instead of *T. salsuginea* in non-taxonomic literature was cast. Note that if the taxa were conspecific, the name *T. salsuginea* should have been applied for the united species as having priority over *T. halophila* and, second, the binominal “*Arabidopsis halophila*” has never been validly published, i.e., it can not be used as a scientific name.

Subsequently, one more closely related species, *T. botschantzevii* D. German, was discovered (German, 2002). The phylogenetic position and relationships of *Thellungiella* was first preliminarily revealed by O'Kane and Al-Shehbaz (2003) who found it closely allied with one member of *Eutrema* R. Br. s. str. and non-monophyletic, if *T. parvula* was included. The item was further elucidated by Warwick et al. (2006) who showed that *Thellungiella* s. str. (without *T. parvula* which was not studied in that work) is monophyletic but is nested within the paraphyletic *Eutrema*. Based on those results, *Thellungiella* was recognized as a congeneric with *Eutrema*, and the latter genus was expanded to accommodate, in particular, all (four) *Thellungiella* representatives, including *T. parvula* [named as *E. botschantzevii* (D. German) Al-Shehbaz and Warwick, *E. halophilum* (C. A. Mey.) Al-Shehbaz and Warwick, *E. parvulum* (Schrenk) Al-Shehbaz and Warwick, and *E. salsugineum* (Pall.) Al-Shehbaz and Warwick] (Al-Shehbaz and Warwick, 2005).

The position of *T. parvula* was then tested in the context of a family-wide phylogeny (German et al., 2009) and its distinct position outside not only *Eutrema* but also the tribe *Eutremeae* Al-Shehbaz, Beilstein and E.A. Kellogg was demonstrated. This finding along with the re-evaluation of the species' morphology, resulted in recognition of a new genus, *Schrenkiella* D. German and Al-Shehbaz, with a single species, thereafter *S. parvula* (Schrenk) D. German and Al-Shehbaz (German and Al-Shehbaz, 2010). Currently *Schrenkiella* is among 20 (out of 321) genera of Brassicaceae who's precise phylogenetic position and tribal affiliation still remains uncertain (Al-Shehbaz, 2012) though its placement within the “core evolutionary lineage II” (Figure 2) which includes, in particular, *Brassica* L., is obvious (some results reveal its closer affinity with the tribe *Thelypodieae* Prantl—R. Schmickl, pers. comm.; see also Cheng et al., 2013).

In parallel to the above taxonomic and phylogenetic clarifications, the data on distribution of three “core” *Thellungiella* species and *S. parvula* were also considerably updated. Thus, *T. botschantzevii*, initially described as endemic to the south-west Siberia, was found in Kazakhstan (German, 2006) and subsequently in Europe (German, 2008). Moreover, it turned out that all previous reports of *T. salsuginea* from Europe belong to *T. botschantzevii* (German, 2008) and distribution of *T. salsuginea* is confined to Asia and North America. Finally, occurrence of both *T. halophila* and *S. parvula* in China was not confirmed where the single *Thellungiella* species, *T. salsuginea*, is documented by herbarium vouchers to date (German and Chen, 2009). Notably, among the discussed species, the most widely mentioned *T. halophila* possesses the narrowest distribution area being restricted to Kazakhstan where other three species do also occur.

Misapplication of various data in relation to *Thellungiella* is thus comparatively wide and extensive. There was some hope that this situation would turn round after the publication of Amtmann (2009) where some of the above discrepancies were briefly highlighted and, indeed, some improvement can be observed in a gradual switching from the name *T. halophila* to *T. salsuginea* (e. g., Lugan et al., 2010; Oh et al., 2010; Orsini et al., 2010; Wu et al., 2012) or *E. salsugineum* (Yang et al., 2013). However, misnomers persist: *T. salsuginea* named as *T. halophila* (e.g., Guo et al., 2012; Lamdan et al., 2012). Moreover, even in those papers where the species name *T. salsuginea* (or *E. salsugineum*) is correctly applied for what is indeed this species, *T. halophila* is often treated now as its synonym (e.g., Oh et al., 2010; Orsini et al., 2010; Pang et al., 2012; Yang et al., 2013), which, as shown above, is completely wrong, and use of the characteristics like “*Thellungiella halophila/salsuginea*” (Ghars et al., 2012) or “*Thellungiella halophila* (Salt cress also known as *Eutrema salsugineum*)” (*Thellungiella halophila* Genome Project, 2011) is equally inappropriate. Therefore, the misunderstanding is continuing, and it is still a very rare case when all three species of true *Thellungiella* are clearly distinguished and their physiological traits are characterized separately (Lee et al., 2012) and real differences in mechanisms of response to the stress factors at the species level are demonstrated (De Boer et al., 2007). This is the only adequate approach to be established in the experimental studies of *Thellungiella*, and brief comparative characteristics of its three closely related species showing some of their morphological characters along with geographic distribution are summarized in Table 2; the data on mating system (self-compatibility versus incompatibility) emphasizing the difference between *T. halophila* and *T. salsuginea* are also included.

**Table 2:** **Brief comparative morphological, geographic and biological characteristics of *Thellungiella* s. str. species**.

**Taxon/character**	***T. salsuginea***	***T. botschantzevii***	***T. halophila***
Rosette leaves	Ovate, margin entire to repand, long petiolate, bright-green, glossy	Round, entire to repand, short petiolate, bright- to dark-green, ± dull	Oblong, pinnatifid to pinnatisect, long petiolate, light-green, glaucous
Stem leaves	Entire, base deeply cordate	Entire, base cordate-sagittate	Entire to pinnate, base cordate to subamplexicaul
Indumentum	Absent	Present (sparse simple trichomes, mostly on lower leaves)	Absent
Seed arrangement in a locule	Biseriate	Uniseriate	Uniseriate
Seed number per fruit	(56)60–100	30–40(44)	16–32
Petals (mm)	2.5–3.7 ×1.0–1.7	2.0–2.5 × 1.0–1.1	2.5–3.5 ×1.5–1.9
Self-compatibility	Compatible^*^	Compatible^*^	Incompatible^*^
Distribution	Asia: China, Kazakhstan, Kyrgyzstan, Russia (Siberia, Far East), America (NW): Canada, USA	Russia (European part, SW Siberia), Kazakhstan	Kazakhstan

* Bert de Boer, pers. comm.

Regarding the genus *Thellungiella* itself (lectotype species *T. salsuginea*), as mentioned before, currently available phylogenetic information indicates its placement within *Eutrema*. Therefore, using the generic name *Thellungiella* implies paraphyletic concept of *Eutrema*. Taking this into consideration, the use of the latter generic name instead of *Thellungiella* is preferable. However, *Thellungiella* clade (within *Eutrema*) is monophyletic with highest support in all relevant studies (O'Kane and Al-Shehbaz, 2003; Warwick et al., 2006; Schmickl, pers. comm.). Consequently, use of the name *Thellungiella* for *T. salsuginea* and two closely related species (not for *S. parvula*) would not severely affect the evolutionary context of relevant studies as soon as it would be applied to the monophyletic group within *Eutremeae*. This is not the case of morphologically and, more important, phylogenetically more distant *S. parvula*.

It should be mentioned that rather recently an attempt to expand *Thellungiella* with another two species, namely *T. pumila* (Steph.) V.I. Dorof. and *T. toxophylla* (M. Bieb.) V.I. Dorof., has been undertaken (Dorofeyev, 2002). Although in the second case this decision has some morphological justification, this is apparently a matter of homoplasy evolved under similar conditions (the species is also halophytic) and the above viewpoint did not get support from any phylogenetic study. Relevant species are currently accepted as *Olimarabidopsis pumila* (Steph.) Al-Shehbaz, O'Kane and Price and *Pseudoarabidopsis toxophylla* (M. Bieb.) Al-Shehbaz, O'Kane and Price from the tribes *Alyssopsideae* Al-Shehbaz, Warwick, Mummenhoff and M. Koch and *Camelineae* DC., respectively (Al-Shehbaz, 2012), both unrelated to *Eutremeae*.

### Conclusions and suggestions on future *Thellungiella* research

1. Generic name *Thellungiella* is currently being applied in genomic and physiological literature to up to four species.
2. Three of these four species (*T. botschantzevii, T. halophila, T. salsuginea*) represent true *Thellungiella* which is monophyletic but is phylogenetically within *Eutrema* (tribe *Eurtemeae*). In order to avoid paraphyletic concept of *Eutrema*, the use of this generic name instead of *Thellungiella* is preferable though using the latter name does not really distort the evolutionary context of the results of relevant studies.
3. All three representatives of true *Thellungiella* are closely related but distinct species which is well supported by their morphology and distribution. Hence, mixing them up (which is especially case for *T. halophila* and *T. salsuginea*) is inappropriate as it would result in producing artifacts. In particular, the name *T. halophila* can not be treated as either the former name or a synonym of *T. salsuginea*. Instead, in all cases of such misapplication it is necessary to clarify which species was in fact studied and specify which name to which species was misapplied (most often the name *T. halophila* was misused for the plants of *T. salsuginea*). Whenever possible, geographic origin of the seed material should be specified as in many cases it can help to verify/confirm identification.
4. Unlike the above species, *T. parvula* can not be treated as a member of either *Thellungiella* or *Eutrema* and represents monospecific genus *Schrenkiella* not very closely related to *Eutremeae*; its phylogenetic position needs further elucidation. For this reason, using the name *T. parvula* as well as *E. parvulum* implies wrong phylogenetic and evolutionary context and should be avoided.

## Conclusions

Biological research is driven by comparative approaches across disciplines (in the widest sense). The choice of *Arabidopsis thaliana* as the first model organism of flowering plants created the first fixed point of reference. Sequencing of its genome a decade ago (The Arabidopsis Genome Initiative 2002), has facilitated genomic comparisons in plants: for annotated genes, structural rearrangements and latterly SNP frequencies among multiple Arabidopsis accessions (1001 Genome Project). Since the advent of this reference genome, many other organisms have become well-established as model systems, allowing in-depth comparative analyses, rice from the monocots within flowering plants for example (Goff et al., 2002; Yu et al., 2002). Prior to the advent of molecular biology in evolutionary research, broadly relevant studies within the Brassicaceae family mainly focused on the characterization and breeding of agronomically important species such as cabbage or rapeseed. The same is true for crucifer systematics and taxonomy (Koch et al., 2003; Koch and Al-Shehbaz, 2009). Taxonomy and systematics predating the advent of molecular marker systems accumulated relatively isolated information whose conclusions were neither taxonomically nor evolutionarily comprehensive.

This gap of comprehensive knowledge created a source of substantial problems for researchers because systematics and taxonomy knowledge is the “access key” to biological information needed for any kind of comparative research. This has been outlined in detail and precisely phrased by Paterson and colleagues' headline (Paterson et al., 2010): “Names are key to the big new biology”. Meanwhile there is no doubt that taxonomic databases do play a central role in providing adequate biological information (e.g., The Plant List, 2010). Such an online information and knowledge tool, *BrassiBase*, (http://brassibase.cos.uni-heidelberg.de/) has been launched recently for Brassicaceae (Koch et al., 2012) with the intention that this bioinformatics tool can be used for the integration of taxonomy, systematics and the evolutionary biology that underpins phylogenetics.

### Conflict of interest statement

The authors declare that the research was conducted in the absence of any commercial or financial relationships that could be construed as a potential conflict of interest.
